# Supplementary material for: A high-resolution mRNA expression time course of embryonic development in zebrafish
Source: eLife. 2017 Nov 16;6:e30860. doi: 10.7554/eLife.30860 (PMC5690287; doi:10.7554/eLife.30860)
Supplement: Supplementary file 6. [file elife-30860-supp6.zip › biolayout-clusters-files/Cluster118-genes.html]

Cluster118


# Cluster118: Genes

| Ensembl ID | Gene Name | Chr | Start | End | Biotype |
| --- | --- | --- | --- | --- | --- |
| ENSDARG00000001909 | VILL | 24 | 20274280 | 20305326 | protein\_coding |
| ENSDARG00000071341 | abch1 | 17 | 24518784 | 24546276 | protein\_coding |
| ENSDARG00000039131 | atp1a1a.3 | 1 | 1657500 | 1667866 | protein\_coding |
| ENSDARG00000076833 | atp1b1b | 1 | 10036183 | 10055110 | protein\_coding |
| ENSDARG00000041595 | ces3 | 18 | 17429188 | 17437240 | protein\_coding |
| ENSDARG00000088484 | kcnj1a.6 | 18 | 48496310 | 48505848 | protein\_coding |
| ENSDARG00000055290 | mpeg1.1 | 8 | 29700878 | 29704389 | protein\_coding |
| ENSDARG00000040306 | otomp | 2 | 41654791 | 41669119 | protein\_coding |
| ENSDARG00000090793 | si:ch211-207j7.2 | 6 | 7844435 | 7858454 | protein\_coding |
| ENSDARG00000098662 | si:ch73-359m17.9 | 19 | 48649356 | 48652715 | protein\_coding |
| ENSDARG00000067797 | spi1a | 25 | 35048603 | 35067306 | protein\_coding |
